# Association between TyG‑BMI and repeat percutaneous coronary intervention (PCI) risk: exploratory stratification by glucose metabolism status

**DOI:** 10.1371/journal.pone.0362761

**Published:** 2026-11-11

**Authors:** Weiqiang Fei, Feng Yang, Yongbang Zhu, YiNa Le, Shan Wang, Danxia Sun, XiaoXu Zhang

**Affiliations:** 1 Hangzhou Polytechnic University, Hangzhou, China; 2 Yipeng Community Health Service Center, Hangzhou, China; Hong Kong Baptist University, HONG KONG

## Abstract

Most patients undergoing percutaneous coronary intervention (PCI) are diagnosed with coronary heart disease (CHD). The triglyceride glucose‑body mass index (TyG‑BMI) has been reported to be associated with CHD. This study aimed to investigate the association between TyG‑BMI and the risk of repeat PCI among post‑PCI patients, with additional exploratory stratification analyses according to glucose‑metabolism status. Data for this study were derived from resident health‑record follow‑up records of Yipeng Community Health Service Center, covering January 2015 to January 2025. Logistic regression models and restricted cubic spline (RCS) were used to evaluate the association between TyG‑BMI and the risk of repeat PCI among post‑PCI patients, with exploratory stratification by glucose‑metabolism status. Logistic regression and restricted cubic spline analyses showed no significant linear or nonlinear association between TyG‑BMI and repeat PCI (*OR*=0.999,*95%CI*:0.994 ~ 1.003, *P* = 0.534; nonlinear *χ*^*2*^ = 0.91, df = 3, *P* = 0.8223). Stratified analyses by glucose metabolism status also revealed no significant correlation in any stratum(*P >* 0.05). No independent association between TyG‑BMI and repeat PCI was identified in post‑PCI patients, even within different glucose‑metabolism strata. Relevant findings require further verification in larger prospective cohorts.

## 1. Introduction

Coronary heart disease (CHD), particularly acute coronary syndrome (ACS), remains a leading cause of cardiovascular mortality and disability globally [1]. The widespread implementation of percutaneous coronary intervention (PCI) and the continuous optimization of dual antiplatelet and lipid‑lowering therapies have substantially improved the short‑term prognosis of patients with CHD [2,3]. A considerable proportion of patients still experience long‑term adverse events after PCI, among which repeat revascularization severely impairs long‑term clinical outcomes [4]. In‑stent restenosis (ISR) and progressive native coronary plaque progression are the predominant drivers of repeat PCI, which places a heavy burden on individual patients and clinical healthcare systems [5]. Accordingly, accurate identification of high‑risk populations vulnerable to repeat PCI is essential for strengthening individualized secondary prevention and improving long‑term prognosis.

Dysregulated glucose and lipid metabolism are well‑established modifiable risk factors for coronary plaque progression and post‑PCI adverse events [6]. Traditional clinical studies have generally explored the prognostic value of single metabolic indicators, such as isolated hyperglycemia or dyslipidemia, while ignoring the comprehensive reflection of overall metabolic status. Composite metabolic biomarkers have emerged as powerful tools for integrated cardiometabolic risk evaluation, as they can reflect multiple metabolic abnormalities simultaneously rather than relying on single metabolic parameters [7]. The triglyceride glucose‑body mass index (TyG‑BMI) is a novel and reliable composite metabolic indicator that has exhibited promising predictive value for cardiovascular diseases [8]. As an integrated marker of insulin resistance and adiposity, TyG‑BMI effectively reflects underlying metabolic disorders and has been closely linked to atherosclerotic progression and adverse cardiovascular events [9]. Existing studies have demonstrated associations between TyG‑BMI and the occurrence of coronary heart disease, yet its predictive role for repeat PCI remains controversial [10].

Few studies have explored the association between TyG‑BMI and repeat PCI risk in post‑PCI patients, and whether this association varies across different glucose‑metabolism states remains unclear. Given that TyG‑BMI reflects insulin resistance and adiposity, this study aimed to investigate the association between TyG‑BMI and repeat PCI risk among post‑PCI patients, with exploratory stratified analyses by glucose‑metabolism status. The results may provide clinical evidence for risk assessment and individualized preventive management for post‑PCI patients.

## 2. Research objects and methods

### 2.1. Research subjects

This was a retrospective case-control study. Through the resident health record data system of community health service center of Yipeng. Data was exported between 01/09/2025 and 30/11/2025. Authors had access to information that could not identify individual participants during or after data collection. Information from the resident health record data system was compiled in abstracted evaluations. Inclusion criteria: (1) Diagnosed with CHD and treated with PCI; (2) Complete health record data. Exclusion criteria: (1) Cases of death in the past decade; (2) Cancer; (3) Long-term medication for immune system diseases. The source population consisted of 2,561 patients with CHD who underwent PCI (01/01/2015 to 01/01/2025). Following application of the inclusion and exclusion criteria, 1,341 patients were excluded, and 1,020 participants were included in the final analysis. Based on the occurrence of repeat PCI as the study outcome, the participants were retrospectively assigned into two groups: the 453 patients who experienced repeat PCI constituted the case group, and the 567 patients who did not constituted the control group. This study followed the basic guidelines of the Declaration of Helsinki and was approved by the Ethics Committee of Hangzhou Ninth People's Hospital (Ethic Approval Number: 2025−008), and the ethics committee approved the waiver of informed consent for all participants. The data collection flow chart is shown in Fig 1.

**Fig 1:**
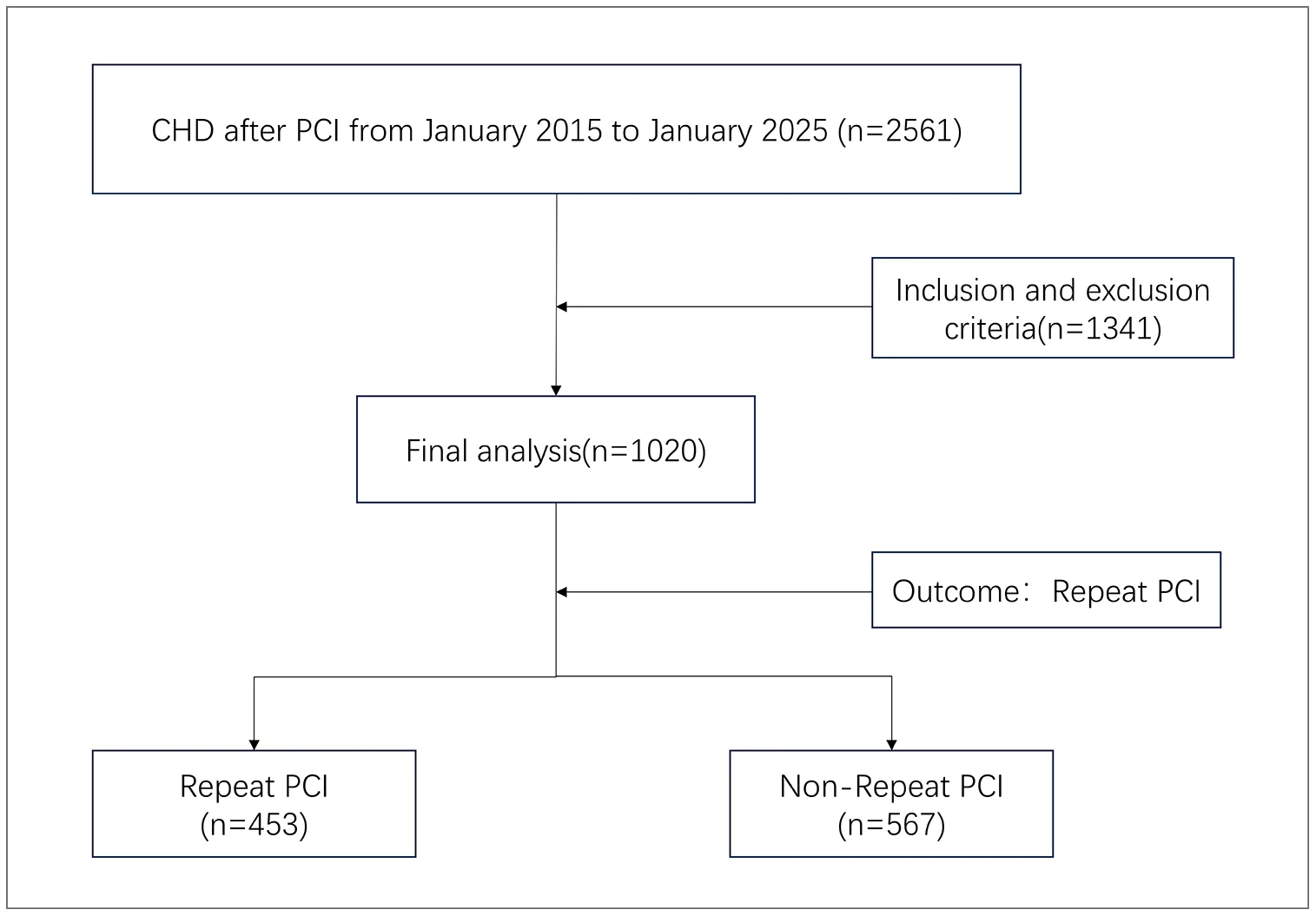
Flow chart.

### 2.2. Data collection and definitions

The health records of residents within the jurisdiction who met the inclusion and exclusion criteria were reviewed. Basic demographic information and relevant laboratory testing data of the research subjects were collected. All data were preoperative data for the first PCI. All the research subjects received dual antiplatelet therapy with oral aspirin 100 mg/day and clopidogrel 75 mg/day or ticagrelor 90 mg/day for at least 1 year after PCI. The research subjects with chronic diseases such as diabetes and hypertension were treated with medicine according to the doctor's advice.

The outcome of repeat PCI was defined as any prior percutaneous coronary intervention performed at any coronary site after the index PCI procedure. This endpoint was not restricted to target‑lesion revascularization (TLR) or target‑vessel revascularization (TVR). Coronary artery bypass grafting (CABG) was not counted as an endpoint event [11,12].


$$\begin{array}{c} BodyMassIndex(BMI)\text{:\textasciitilde{}}BMI=weight(kg)/height\text{\textasciitilde{}}(m)2.\text{\textasciitilde{}} \end{array}$$

$$\begin{array}{c} TyG=ln[TG(mg/dl)\times FBG(mg/dl)/2] \end{array}$$

$$\begin{array}{c} TyG-BMI=ln[TG(mg/dl)\times FBG(mg/dl)/2]\times BMI \end{array}$$

Glucose‑metabolism status was classified at baseline before the index PCI according to Chinese Guidelines for the Prevention and Treatment of Type 2 Diabetes (2024) [13]. (1) Diabetes group: patients with prior physician‑diagnosed diabetes; or patients without diabetes history with HbA1c≥6.5%. For subjects with non‑diabetic‑range HbA1c but markedly elevated baseline fasting blood glucose (FBG), FBG was only used for clinical reference and would not alter group assignment. (2) Prediabetes group: no prior diabetes history, with 5.7% ≤ HbA1c < 6.5%. (3) Normal glucose‑metabolism group: no prior diabetes history, with HbA1c < 5.7%. FBG was only applied for TyG‑BMI calculation and served merely as auxiliary reference for stratification, with no independent cutoff values for group assignment. Oral glucose tolerance test (OGTT) data were not available in this dataset.

### 2.3. Statistical analysis

Data were organized using Microsoft Excel, and statistical analyses were performed with SPSS 25.0 and R 4.6.0. Normally distributed measurement data were expressed as mean ± standard deviation, and the independent samples *t* test was used for inter‑group comparisons. Non‑normally distributed measurement data were presented as medians (25th, 75th percentiles), and the non‑parametric rank sum test was adopted for comparisons of medians between groups. Categorical data were reported as n (%), and the χ² test was used to compare proportions across groups. Fisher’s exact test was applied when the assumptions for the χ² test were not met. Multivariate logistic regression models were fitted to calculate odds ratios (OR) and their corresponding 95% confidence intervals (95%CI). Restricted cubic spline(RCS) plots were utilized to explore the non‑linear relationships between variables. A two‑tailed P‑value < 0.05 was considered statistically significant.

## 3. Results

### 3.1. The association between baseline characteristics and repeat PCI

A total of 1020 participants were included in this study. The overall mean age was 62.31 ± 10.53 years. Among all participants, 453 (44.4%) underwent repeat PCI, and 49.1% of the total participants were male. Smoking and most clinical indicators (TC, TG, LDL‑C, HDL‑C, BNP, cTnT, Lp‑a, D‑D, eGFR) showed statistically significant inter‑group differences between patients with and without repeat PCI (*P* < 0.05), although the absolute between‑group differences were small, suggesting limited clinical relevance. Table 1 for details.

**Table 1:** Analysis of the association between baseline characteristics and repeat PCI (*n* = 1020).

Variables	Repeat PCI		*t/χ* ^*2*^ */Z*	*P*-value
	No	Yes		
Age (years)	62.47 ± 10.17	62.08 ± 10.93	0.602	0.547
Sex (Men %)	291(58.3)	208(41.7)	2.946	0.086
Hypertension(%)	377(54.0)	321(46.0)	2.227	0.136
Stroke(%)	49(53.3)	43(46.7)	0.222	0.638
Smoking(%)	163(47.7)	179(52.3)	13.097	**< 0.001**
Drinking(%)	142(54.8)	117(45.2)	0.082	0.775
TC(mmol/L)	3.21 ± 1.36	3.51 ± 1.91	−2.886	**0.006**
TG(mmol/L)	1.37 ± 0.79	1.54 ± 0.89	−3.502	**0.002**
LDL-C(mmol/L)	1.72 ± 0.57	2.00 ± 0.78	−6.183	**< 0.001**
HDL-C(mmol/L)	1.11 ± 0.33	1.03 ± 0.29	4.111	**< 0.001**
BNP(pg/ml)	82.40(39.90,136.90)	94.50(44.50,185.20)	−2.697	**0.007**
cTnT(µg/L)	0.01(0.01,0.02)	0.01(0.01,0.03)	−5.234	**< 0.001**
Lp-a(mmol/L)	13.9(6.62,37.25)	21.18(7.25,50.51)	−2.758	**0.006**
D-D(ug/ml)	0.26(0.21,0.37)	0.29(0.20,0.47)	−2.447	**0.014**
APTT(s)	35.61 ± 5.30	35.13 ± 6.77	1.426	0.154
eGFR(ml/min/1.73m^2^)	93.35 ± 26.29	98.08 ± 34.93	−2.771	**0.007**

### 3.2. The association between TyG-BMI and repeat PCI

TyG was significantly different between the two groups in terms of whether they underwent repeat PCI (*P <* 0.05), whereas TyG-BMI was not(*P >* 0.05). Table 2 for details.

**Table 2:** Analysis of the association between TyG-BMI and repeat PCI (*n* = 1020).

Variables	Repeat PCI		*t*	*P*-value
	No	Yes		
TyG	7.08 ± 0.60	7.23 ± 0.66	−2.943	**0.003**
BMI	26.06 ± 3.43	26.10 ± 3.41	−0.362	0.718
TyG-BMI	185.17 ± 31.49	189.17 ± 33.10	−1.373	0.171

### 3.3. Logistic regression and RCS analysis

Multivariate logistic regression was used to adjust for confounders and explore the independent association between continuous TyG‑BMI and the risk of repeat PCI, where the outcome was defined as the occurrence of repeat PCI. The variable inclusion strategy for the model was as follows: variables with *P* < 0.05 in univariate analysis were included in the candidate model. To adequately control for demographic confounding, age and gender were forcibly retained in the final model irrespective of P‑values. Among lipid parameters, only HDL and LDL were kept. TG was excluded to avoid multicollinearity with TyG‑BMI, since TG constitutes a core component of the TyG index. As a composite summary measure of lipid fractions, TC shows strong intrinsic correlations with TG, HDL and LDL and was therefore also excluded. A backward stepwise elimination procedure was applied for model optimization, with a variable entry criterion of *P* < 0.10 and a removal criterion of *P* ≥ 0.05. With continuous TyG‑BMI as the primary exposure variable, odds ratios (OR) and 95% confidence intervals (95% CI) were calculated to quantify its independent association with repeat PCI. In addition, RCS was performed to assess the nonlinear dose‑response relationship between TyG‑BMI and repeat PCI risk. Knots were placed at the 5%, 27.5%, 50%, 72.5%, 95%percentiles of the TyG‑BMI distribution, and the likelihood‑ratio test was used to verify the presence of a nonlinear association. The variable‑selection strategy for the model was the same as described above.

After adjustment for confounding variables, the logistic regression indicated that TyG-BMI was not an independent influencing factor for repeat PCI(*OR*=0.999,*95%CI*:0.994 ~ 1.003, *P* = 0.534), Fig 2 for details. RCS showed that no significant linear association was observed between continuous TyG-BMI and the risk of repeat PCI (overall *χ*^*2*^ = 1.31, df = 4, *P* = 0.8589). Further nonlinearity test confirmed that the dose‑response relationship did not exhibit a statistically significant nonlinear trend (nonlinear *χ*^*2*^ = 0.91, df = 3, *P* = 0.8223), Fig 3 for details.

**Fig 2:**
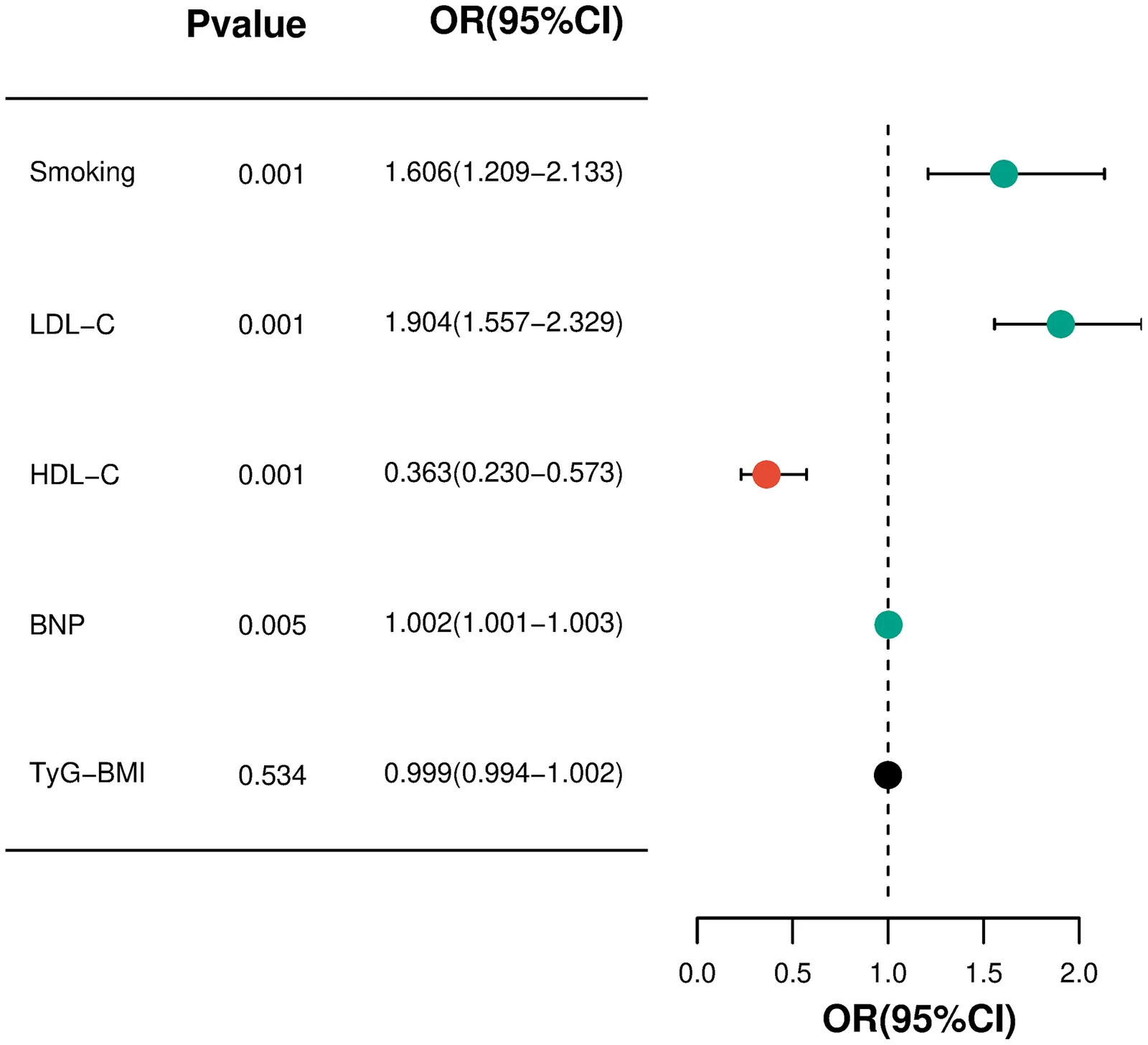
Forest plot of logistic regression. * Model diagnostics for the multivariable *logistic regression: Hosmer‑Lemeshow test (P* = *0.164), variance inflation factor (all VIF < 5), and* AUC = *0.562 (95%CI: 0.511–0.643).*

**Fig 3:**
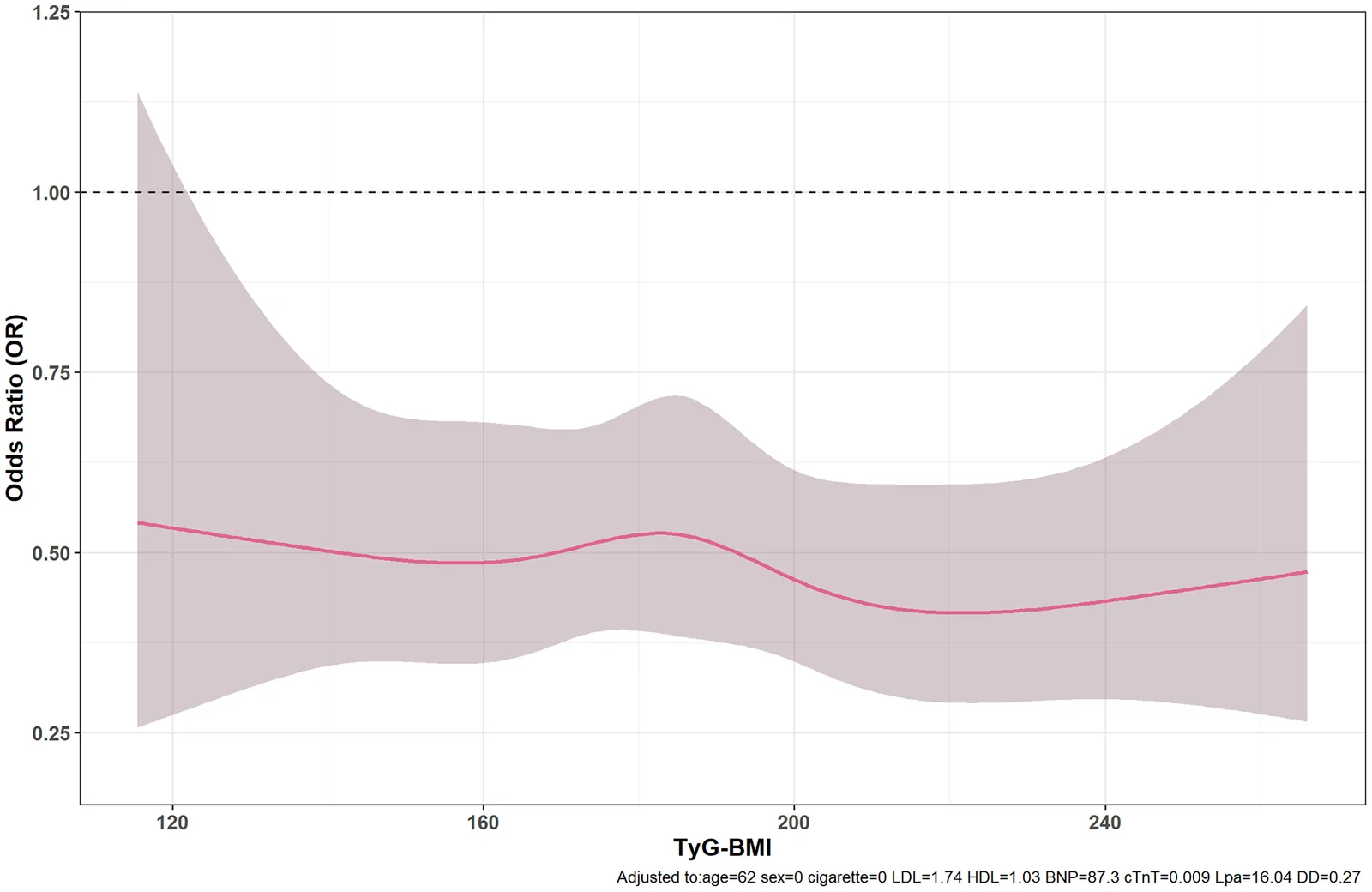
Restricted cubic spline plot.

### 3.4. The association between TyG‑BMI and repeat PCI according to different glucose metabolism states

Among the 1020 participants, 160(15.7%) were normoglycemia, 489(47.9%) were prediabetes, 371(36.4%) were diabetes. There was no statistically significant difference in the proportion of patients who underwent repeat PCI among the three groups(*P >* 0.05). Fig 4 for details.

**Fig 4:**
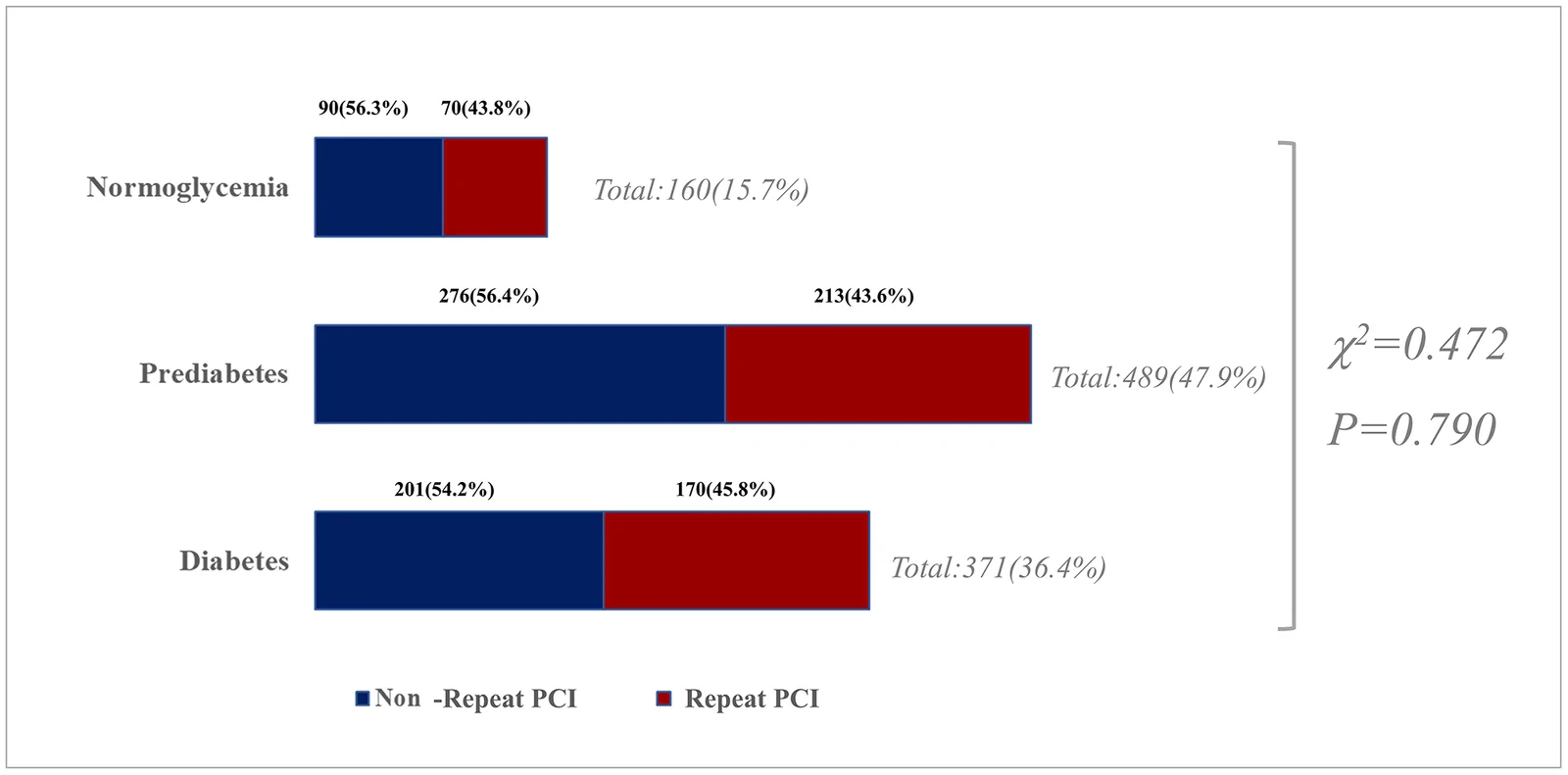
Analysis of the association between different glucose metabolism states and repeat PCI.

Stratified analyses according to glucose metabolism levels were performed to explore the association between TyG-BMI and repeat PCI. No statistically significant association was observed between TyG-BMI and repeat PCI within any stratum. Table 3 for details.

**Table 3:** Analysis of the association between TyG-BMI and repeat PCI in different glucose metabolism states (*n* = 1020).

Variables	Repeat PCI		*t*	*P*-value
	No	Yes		
**Total**				
TyG-BMI	185.17 ± 31.49	188.81 ± 32.76	−1.787	0.074
**Normoglycemia**				
TyG-BMI	172.68 ± 27.85	180.24 ± 26.46	−1.750	0.082
**Prediabetes**				
TyG-BMI	183.41 ± 29.47	187.34 ± 33.26	−1.360	0.174
**Diabetes**				
TyG-BMI	193.19 ± 33.57	194.16 ± 33.69	−0.277	0.782

## 4. Discussion

This study showed that there was no significant association between baseline TyG‑BMI and the risk of repeat PCI during follow‑up among patients with CHD undergoing PCI. When TyG‑BMI was included as a continuous variable in the logistic regression model and the dose‑response relationship was assessed using RCS, neither obvious linear nor nonlinear associations were observed. After stratified analysis according to glucose metabolism status (normoglycemia, prediabetes, diabetes), no statistically significant association between TyG‑BMI and repeat PCI was found within each stratum. These findings suggest that TyG‑BMI may not be an influencing factor for repeat PCI in CHD, and its use for evaluating risk factors for repeat PCI after PCI is not supported.

Previous studies have shown that increased TyG-BMI is closely associated with the incidence of CHD, severe coronary lesions, post-PCI adverse cardiovascular events and repeat revascularization [14,15]. However, our study found that TyG-BMI was not independently associated with the risk of repeat PCI during follow-up among patients with CHD who had undergone primary PCI. This may be attributed to the fact that PCI procedure itself and standardized secondary preventive therapy after PCI alter the natural history of CHD, reducing the predictive ability of baseline metabolic indicators for long-term events [16,17]. Most prior studies included patients with incident CHD [18], ACS [19,20], or patients without prior revascularization. In these populations with naturally progressing coronary lesions, the association between the composite metabolic marker TyG-BMI and coronary events was relatively clear. All patients in our study had undergone primary PCI, which significantly improved myocardial perfusion, and received standardized secondary preventive pharmacotherapy after the intervention. Although TyG-BMI was calculated based on preoperative baseline FBG, TG and BMI, the above interventions substantially reduced the overall risk of recurrent coronary events, diluting or even eliminating the original association between preoperative baseline metabolic abnormalities and long-term postoperative outcomes. Furthermore, the decision for repeat PCI arises from not a single cause but multidimensional pathophysiological processes, such as ISR [21], stent thrombosis(ST) [22], progression of original non-target lesions, and newly developed coronary atherosclerotic lesions [23,24]. TyG-BMI represents only one of the numerous driving factors, with a limited total attributable weight. Consequently, it failed to reach statistical significance in both univariate analysis and multivariate-adjusted models.

Stratified analyses by different glycemic metabolic statuses showed no significant association between TyG-BMI and repeat PCI. Glycometabolic disturbance and IS are well-recognized important drivers of coronary atherosclerosis progression and ISR [25]. The burden of coronary artery disease is markedly higher in patients with diabetes than in those without diabetes [26]. The risk of coronary events is expected to increase in a stepwise manner across three groups: normoglycemia, prediabetes, and diabetes, and the incidence of repeat PCI should exhibit a corresponding gradient difference [27]. Risk of coronary events rises stepwise in patients with normal glucose, prediabetes and diabetes, with a corresponding gradient in the rate of repeat PCI. However, we did not observe this anticipated trend in our study. All study participants were post-first PCI patients, and all received standardized secondary prevention therapy. Post-PCI patients had a high coverage of standardized medication. All patients received relatively homogeneous intensive interventions after the procedure. The additional metabolic risk among individuals with prediabetes and diabetes was partially offset by pharmacotherapy, substantially narrowing the residual risk difference across the three groups and leading to similar rates of repeat PCI. Post-PCI patients had a high coverage of standardized medication. All patients received relatively homogeneous intensive interventions after the procedure. The additional metabolic risk among individuals with prediabetes and diabetes was partially offset by pharmacotherapy, substantially narrowing the residual risk difference across the three groups and leading to similar rates of repeat PCI.

## 5. Conclusion

Among patients with CHD who underwent primary PCI and received standardized secondary prevention, baseline TyG‑BMI was not independently associated with the risk of repeat PCI during follow‑up, and no association was observed across strata of different glycemic metabolic statuses. The PCI procedure itself and post‑procedural standardized pharmacotherapy alter the natural course of CHD. Meanwhile, repeat PCI represents a multifactorial‑driven composite endpoint, which attenuates the crude association between baseline metabolic composite indices and long‑term clinical outcomes. For clinical management after PCI, TyG‑BMI should not be adopted as a risk‑stratification tool for repeat PCI. Clinical decision‑making should rely on dynamic monitoring of traditional risk factors and imaging assessments. Future studies are warranted to further clarify the optimal application scenarios of TyG‑BMI in distinct CHD groups.

## 6. Strengths and limitations

Since this study only included patients who survived after primary PCI and excluded individuals with in‑hospital death following PCI, survivor bias may exist. This could bias the effect estimates and partially limit the generalizability of our findings. Secondly, the database of this study lacks information regarding stent type, lesion complexity, SYNTAX score, medication adherence, and inflammatory markers, leading to residual confounding related to PCI associated factors. Repeat PCI is affected by coronary anatomical characteristics and procedural factors, which may mask the potential association between TyG‑BMI and repeat PCI. This also provides a plausible explanation for the null findings observed in our study.
